# Large Scale Spatial Risk and Comparative Prevalence of *Borrelia miyamotoi* and *Borrelia burgdorferi* Sensu Lato in *Ixodes pacificus*

**DOI:** 10.1371/journal.pone.0110853

**Published:** 2014-10-21

**Authors:** Kerry Padgett, Denise Bonilla, Anne Kjemtrup, Inger-Marie Vilcins, Melissa Hardstone Yoshimizu, Lucia Hui, Milagros Sola, Miguel Quintana, Vicki Kramer

**Affiliations:** 1 California Department of Public Health, Vector-Borne Disease Section, Richmond, California, United States of America; 2 Public Health Command Region-West, Joint Base Lewis-McChord, Washington, United States of America; University of Kentucky College of Medicine, United States of America

## Abstract

*Borrelia miyamotoi* is a newly described emerging pathogen transmitted to people by *Ixodes* species ticks and found in temperate regions of North America, Europe, and Asia. There is limited understanding of large scale entomological risk patterns of *B. miyamotoi* and of *Borreila burgdorferi* sensu stricto (ss), the agent of Lyme disease, in western North America. In this study, *B. miyamotoi*, a relapsing fever spirochete, was detected in adult (n = 70) and nymphal (n = 36) *Ixodes pacificus* ticks collected from 24 of 48 California counties that were surveyed over a 13 year period. Statewide prevalence of *B. burgdorferi* sensu lato (sl), which includes *B. burgdorferi* ss, and *B. miyamotoi* were similar in adult *I. pacificus* (0.6% and 0.8%, respectively). In contrast, the prevalence of *B. burgdorferi* sl was almost 2.5 times higher than *B. miyamotoi* in nymphal *I. pacificus* (3.2% versus 1.4%). These results suggest similar risk of exposure to *B. burgdorferi* sl and *B. miyamotoi* from adult *I. pacificus* tick bites in California, but a higher risk of contracting *B. burgdorferi* sl than *B. miyamotoi* from nymphal tick bites. While regional risk of exposure to these two spirochetes varies, the highest risk for both species is found in north and central coastal California and the Sierra Nevada foothill region, and the lowest risk is in southern California; nevertheless, tick-bite avoidance measures should be implemented in all regions of California. This is the first study to comprehensively evaluate entomologic risk for *B. miyamotoi* and *B. burgdorferi* for both adult and nymphal *I. pacificus*, an important human biting tick in western North America.

## Introduction

The relapsing fever spirochete, *Borrelia miyamotoi*, has recently been identified as a human pathogen in Russia, the Netherlands, Japan, and northeastern United States and is now considered an emerging infectious disease [1]–[6]. *Borrelia miyamotoi* was first identified in 1995 in *Ixodes persulcatus* ticks and blood from a Japanese field mouse (*Apodemus argenteus*) from the northern island of Japan [7]. *Borrelia miyamotoi* has since been detected in *Ixodes* tick species from other regions, including *I. ricinus* in Europe, *I. scapularis* in eastern North America [7]–[15], *I. dentatus* in mid-western North America [16], and *I. pacificus* in western North America [17]–[19]. *Borrelia miyamotoi* is one of three relapsing fever *Borrelia* species associated with ixodid ticks, along with *B. lonestari* [20] and *B. theileri* [21], [22]. Possible vertebrate reservoir hosts in North America include small rodents such as the white-footed mouse, deer, and ground dwelling birds such as wild turkeys [13], [16], [23], [24]. Unlike *B. burgdorferi* sensu stricto (ss), the agent of Lyme borreliosis, *B. miyamotoi* spirochetes can be maintained in ticks via transovarial and transtadial transmission and therefore do not necessarily require a vertebrate reservoir host to maintain the infection [8], [13], [25].

In both North America and Europe several members of the *B. burgdorferi* sensu lato complex, which includes *B. burgdorferi* ss, and *B. miyamotoi* have overlapping tick vectors and reservoir host species [13]. The prevalence of *B. burgdorferi* sensu lato (sl) in northwestern California *I. pacificus* nymphs averages 5%, [26] with prevalence locally as high as 20–40% [27], [28]. In southern California, the prevalence of *B. burgdorferi* sl in nymphal *I. pacificus* is much lower, generally less than 0.5% [29]. In a study conducted in the northeastern United States, *B. burgdorferi* ss was found to be 10 times more prevalent than *B. miyamotoi* in *I. scapularis* nymphs (20% versus 2%), and twice as prevalent in rodent reservoirs (12% versus 6%) [13]. In northern California (Mendocino County), the prevalence of *B. miyamotoi* in *I. pacificus* ticks has been detected at a prevalence similar to the Northeast in nymphs (1.7%) and slightly less in adults (0.7%) [18]. *Ixodes pacificus* is found throughout California, except in desert and high mountain regions. Like *I. scapularis, I. ricinus,* and *I. persulcatus, I. pacificus* adults and nymphs readily bite people.

Similar to other relapsing fever group spirochetes, *B. miyamotoi* is not easy to culture. Although the original isolates were cultured from tick and rodent blood [7], subsequent efforts to culture spirochetes from ticks or from human case patients have been unsuccessful in other regions of the world [14], [30]. For suspected human cases of tick-borne relapsing fever, detection by blood smear is the standard diagnostic technique [31]. Two human cases of *B. miyamotoi* were diagnosed by direct detection of spirochetes in cerebrospinal fluid and follow-up polymerase chain reaction (PCR) [3], [4]. All other human cases have been diagnosed based on molecular detection of *B. miyamotoi* DNA in acute whole blood from patients [1], [6].

Recent studies have linked previously considered “non-pathogenic” bacterial species or viruses initially described from ticks to human illness, including *B. bissettii* [32] and *Rickettsia philipii* [33] in California, *R. parkeri* in southeastern North America [34], and deer tick virus in eastern North America [35] ^.^ The clarification of emerging tick-borne pathogen ecology, especially in the context of comparison to what we know about *B. burgdorferi* sl, provides an important contextual basis from which public health response and messaging can benefit. As part of on-going tick surveillance for *B. burgdorferi* sl in California, testing for *B. miyamotoi* was added to the statewide tick testing program. This additional surveillance enhances our understanding of *B. miyamotoi* ecology by determining the geographic range and prevalence in California.

## Materials and Methods

### Tick collections

From 2000 through 2012, California Department of Public Health (CDPH) staff and partner agencies collected *Ixodes pacificus* adults and nymphs, primarily in recreational areas, such as forest service campgrounds, hiking trails or picnic areas in national, state, or regional parks. No permit is required for CDPH staff or staff working under CDPH supervision to collect ticks in California per the California Health and Safety Code 116110–116112. These studies did not involve any endangered or protected species. Ticks were collected from vegetation, leaf litter, or other substrate (e.g., logs, tree trunks, rocks), using 1-meter^2^ white double nap flannel “flag” attached to a 1.5-meter wooden dowel. Ticks were either maintained alive within 10#dram (37 mL) polystyrene containers (Fisher Scientific, USA) retained in sealed plastic bags with moistened paper toweling at 3°C (adults) or retained in 70% ethanol (subset of nymphs). In lieu of sampling a prescribed area, collectors typically flagged for a minimum of an hour and submitted at least 20 ticks per 779 collection events; many collections were opportunistic and most were at novel locations. These collections are posted on a California statewide interactive map: http://cdphgis.maps.arcgis.com/apps/SocialMedia/index.html?appid=8d99fb1135d1424f9d8a8711acb7d459.

### Tick testing protocols

Ticks were tested by one of two protocols: A) pools of up to ten ticks were tested by PCR, using primers targeting generic *Borrelia* followed by *B. burgdorferi* specific primers or B) individually tested by using fluorescein-labeled antibody to detect *Borrelia* species spirochetes visually with positive ticks subjected to follow-up nested PCR testing with primers that differentiated between *B. burgdorferi* sl group and relapsing fever group *Borrelia* [36]. Both protocols are described in detail below.

From 2000 to 2009, ticks were tested in pools as follows. Live or frozen *I. pacificus* adults or nymphs were submitted to the US Army, Public Health Command Region–West, Washington for testing. Up to ten ticks of the same sex, stage, and collection site were placed in tubes for DNA extraction. Total DNA was extracted from the pooled ticks using the IsoQuick Nucleic (Orca) Acid Extraction kit (MicroProbe Corp, Bothell, WA, USA). Initially, a nested PCR protocol that targets the flagellin (*fla*) gene (GenBank Accession number: X69611) was used to screen samples; this protocol was *Borrelia* generic [37]. The PCR assays were performed using the illustra PuReTaq Ready-T-Go PCR Beads system (GE Healthcare Biosciences, Pittsburg, PA, USA) under the following conditions: 30 seconds at 95°C, 30 seconds at 55°C, and 1 minute at 75°C. Both external and internal nested PCR reactions were run for 40 cycles. *Borrelia burgdorferi* (B31) DNA isolated from culture was used as positive controls. *Borrelia* positive tick pools were further screened with one of two additional primer sets that were species-specific for *B. burgdorferi* sl spirochetes. These primers include sets developed to amplify a segment of the P66 gene [38] and species-specific flagellin primers [39] where positive results indicated *B. burgdorferi* sl infection. If negative for these *B. burgdorferi* sl-specific primers, a 614 bp partial flagellin gene sequence was compared to selected *Borrelia* sequences (e.g., *B. miyamotoi*) in Genbank (using Clustal X,1.81) to determine species. Results from this testing protocol are stated as minimum infection prevalence (MIP) per stage and by location: (Number of positive tick pools/number of total ticks tested) × 100.

Beginning in 2007 the tick testing protocol was modified to test live individual ticks. *Ixodes pacificus* were tested by CDPH Vector-Borne Disease Section laboratory using a direct fluorescent antibody assay (DFA) using fluorescein-labeled *Borrelia* generic antibodies (Kirkegaard & Perry Laboratories, Inc, Gaithersburg, MD, USA) as previously described [36], [40]. For those ticks with visible spirochetes detected by DFA, DNA was extracted from reserved frozen tissue using the DNeasy Blood and Tissue kit (Qiagen, Germantown, MD, USA) and a nested PCR was performed. The first reaction of the nested PCR targeted the 16S–23S rRNA intragenic spacer region was used to screen for the presence of *Borrelia* (1,336 bp) and was followed by a nested reaction that separated *B. burgdorferi* sl (970 bp) from *B. miyamotoi* (450 bp) [40]. Each PCR reaction mix included 5 µl of extracted tick DNA amplified in a 50 µl reaction mix containing 5 µM of each primer, 5 µl of 10X Thermopol Buffer (New England Biolabs, Ipswich, MA, USA), 1 µl dNTP solution (New England Biolabs, Ipswich, MA, USA), 0.25 µl *Taq* DNA polymerase (New England Biolabs, Ipswich, MA, USA) and autoclaved sterile Milli-Q water. For each reaction, a negative control of autoclaved Milli-Q water was used and a pure DNA isolate of *B. burgdorferi* ss strain B31 antigen was used as a positive control. PCR products were separated using the E-gel agarose gel electrophoresis system on pre-cast 2% gels stained with SYBR safe (Invitrogen, Carlsbad, CA, USA). Results from this testing protocol are expressed as prevalence: (the number of positive ticks/number of ticks tested) × 100. Starting in 2007, a subset of ticks were screened with DFA and positive samples tested by nested PCR; from 2010–2012 all ticks were tested with this protocol.

**Figure 1:**
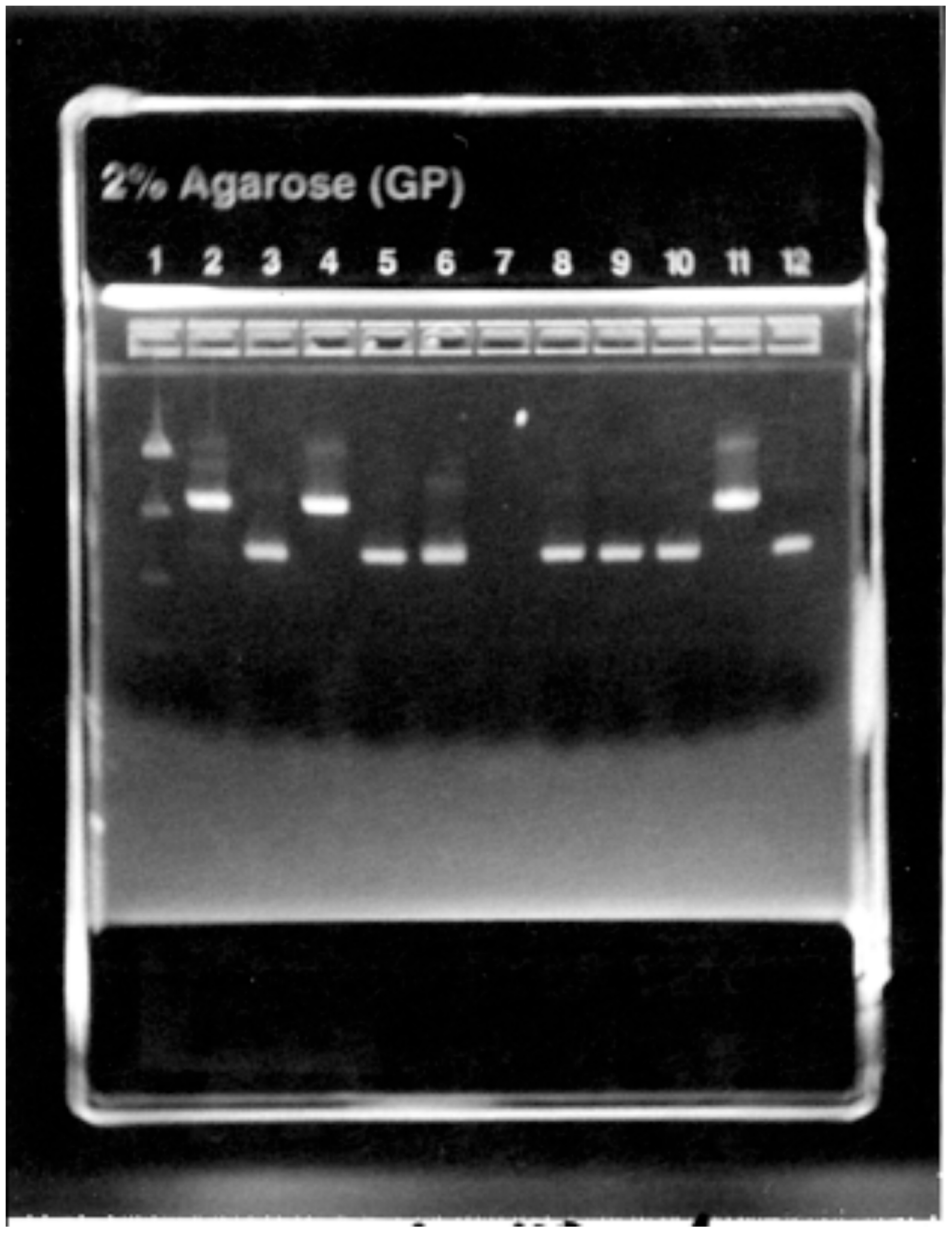
Partial 16S–23S rRNA intragenic spacer region of *Borrelia miyamotoi* (450 bp) and *B. burgdorferi* sl (970 bp).

All ticks that tested positive for *Borrelia* species by either *Borrelia*-generic PCR or by DFA were then subsequently characterized by either comparative sequence analysis or by distinct gel sizes. All positive ticks were found to be either *B. burgdorferi* sl or *B. miyamotoi* (Figure 1). Since the initial focus of the study was to ascertain *B. burgdorferi* prevalence, a subset of ticks was tested only to *Borrelia* genus level after testing negative for *B. burgdorferi* sl. Ticks that tested positive for *B. burgdorferi* were not further tested to specific genomospecies and are considered *B. burgdorferi* sl.

## Results

During 2000–2012, California Department of Public Health (CDPH) staff and partner agencies collected 24,635 adult and 3,252 nymphal *I. pacificus* from 47 counties in 779 separate collection events/sites (Table S1). From 2000 to 2009, the following *I. pacificus* were tested in pools of up to 10 ticks per pool: 18,599 adults in 2,184 pools and 1,064 nymphs in 110 pools from 34 counties (Table 1). Of the pooled ticks tested with the first protocol using PCR with flagellin primers, 108 pools of the adult *I. pacificus* were positive for *Borrelia* spirochetes (0.6% minimum infection prevalence (MIP); n = 2,184 pools of 18,599 ticks). Of these positive pools, 61 pools (0.3% MIP) were positive for *B. burgdorferi* sl and 19 (0.1% MIP) were positive for *B. miyamotoi*. Although the remaining 28 pools were not positive for *B. burgdorferi* sl, they were not sequenced due to lack of material. Of the 1,064 nymphal *I. pacificus* tested in 110 pools, 15 (1.4% MIP) of these pools were positive for *Borrelia* spirochetes. *Borrelia burgdorferi* sl was detected in 9 nymphal tick pools (0.9% MIP) and *B. miyamotoi* in 6 pools (0.6% MIP).

**Table 1:** California *Ixodes pacificu*s adult and nymphal ticks, tested in pools or tested individually, 2000–2012.

	No. Ticks Tested (in No. Pools) 2000–2009		No. Ticks Tested Individually 2007–2012	
County	Adult Ticks Tested(in Pools)	Nymphal Ticks Tested(in Pools)	Adult Ticks TestedIndividually	Nymphal Ticks TestedIndividually
Alameda	0	0	73	0
Alpine	0	0	0	0
Amador	136 (16)	0	95	23
Butte	431 (48)	0	58	161
Calaveras	258 (38)	0	214	12
Colusa	0	0	5	0
Contra Costa	5 (2)	152 (17)	952	218
Del Norte	37 (7)	0	0	0
El Dorado	613 (73)	11 (3)	312	82
Fresno	30 (6)	0	2	0
Glenn	0	0	11	0
Humboldt	54 (7)	0	31	2
Imperial	0	0	0	0
Inyo	0	0	0	0
Kern	0	0	2	0
Kings	0	0	0	0
Lake	685 (76)	23 (5)	449	319
Lassen	0	0	0	0
Los Angeles	5061 (546)	2 (1)	476	3
Madera	145 (19)	0	0	2
Marin	0	0	406	240
Mariposa	84 (14)	0	106	3
Mendocino	0	0	54	17
Merced	0	0	0	0
Modoc	0	0	0	0
Mono	0	0	0	0
Monterey	561 (79)	0	49	2
Napa	99 (10)	0	1	101
Nevada	108 (15)	24 (4)	47	117
Orange	0	0	81	0
Placer	1718 (179)	33 (3)	10	39
Plumas	53 (8)	0	0	0
Riverside	1299 (187)	0	379	1
Sacramento	0	0	0	37
San Benito	0	0	47	4
San Bernardino	286 (34)	0	0	0
San Diego	24 (3)	0	23	0
San Francisco	0	0	0	0
San Joaquin	22 (8)	0	0	0
San Luis Obispo	230 (37)	0	0	0
San Mateo	0	0	316	39
Santa Barbara	716 (81)	0	0	0
Santa Clara	449 (47)	79 (7)	167	131
Santa Cruz	428 (57)	0	752	443
Shasta	1261 (133)	0	389	3
Sierra	0	0	12	0
Siskiyou	37 (4)	0	16	0
Solano	0	0	142	0
Sonoma	928 (113)	570 (59)	53	159
Stanislaus	63 (13)	0	107	0
Sutter	0	0	0	0
Tehama	4 (2)	0	0	0
Trinity	1681 (184)	3 (1)	56	0
Tulare	698 (82)	4 (2)	0	0
Tuolumne	268 (41)	163 (11)	12	3
Ventura	127 (15)	0	0	0
Yolo	0	0	0	0
Yuba	0	0	131	27
**Totals**	**18599 (2184)**	**1064 (110)**	**6036**	**2188**

The non-*B. burgdorferi* sl positive ticks were sequenced for the partial flagellin gene (614 bp) and BLAST analysis indicated that they most closely aligned with *B. miyamotoi* (96.9%) (GenBank accession number: AY024344.1) and more distantly with *B. lonestari* (91.0%). The non-*B. burgdorferi* sl positive ticks were also found to be more distantly aligned with *B. burgdorferi* sl (86.4%), *B. garinii* (86.5%) and *B. afzelii* (82.2%).

The prevalence as calculated with the individual tick-testing approach, provided higher prevalence estimates compared to the minimum infection prevalence pool testing approach. Beginning in 2007, the tick testing protocol was modified to test individual ticks. *Ixodes pacificus* (6,036 adults and 2,188 nymphs) from 38 counties were tested individually (Table 1), screened first by DFA, with *Borrelia* positives further characterized by nested PCR. Parallel to results of ticks tested in pools, *B. burgdorferi* sl and *B. miyamotoi* had similar prevalence in adult ticks with 37 individual ticks (0.6%) positive for *B. burgdorferi* sl and 51 (0.8%) positive for *B. miyamotoi.* Interestingly, nymphal *I. pacificus* infection prevalence was 2.5 times higher for *B. burgdorferi* sl (3.2%) than for *B. miyamotoi* (1.4%) (Table 2). A single nymph from Marin County tested positive for both *B. burgdorferi* sl and *B. miyamotoi*. While both DFA and nestedPCR are sensitive assays for detection of *Borrelia,* the inherent limitation of all diagnostic pathogen detection methods underestimate infection prevalence.

**Table 2:** Proportion of individual adult and nymphal *Ixodes pacificus* ticks with *Borrelia burgdorferi* sensu lato and *B. miyamotoi* detected in California, 2009–2012.

Stage	*B. burgdorferi* s.l.,% (No.)	*B. miyamotoi* % (No.)	N
*I. pacificus* adults	0.6% (37)	0.8% (51)	6036
*I. pacificus* nymphs	3.2% (70)	1.4% (30)	2188

**Figure 2:**
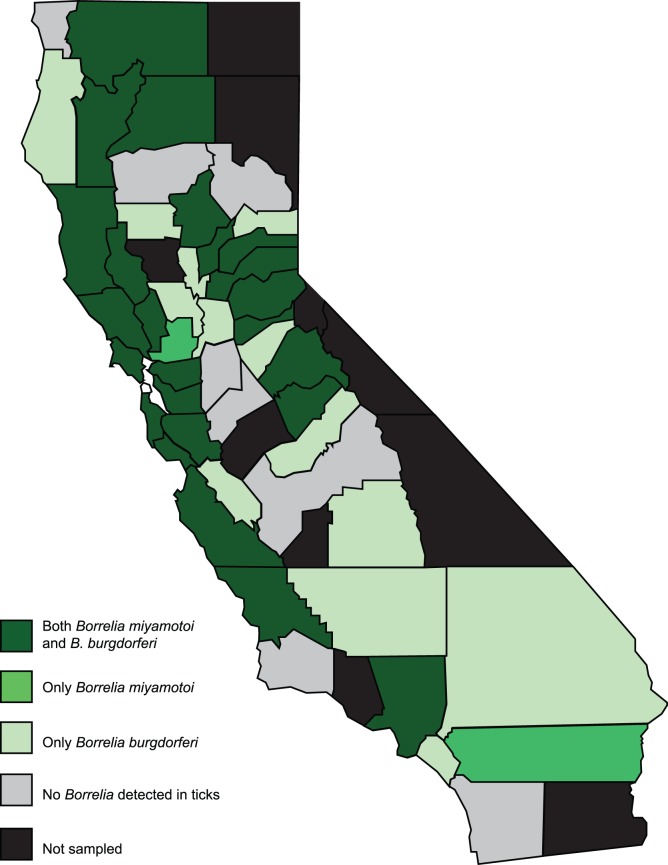
*Borrelia burgdorferi* sl and/or *B. miyamotoi* detected by California counties, 2000–2012.

**Figure 3:**
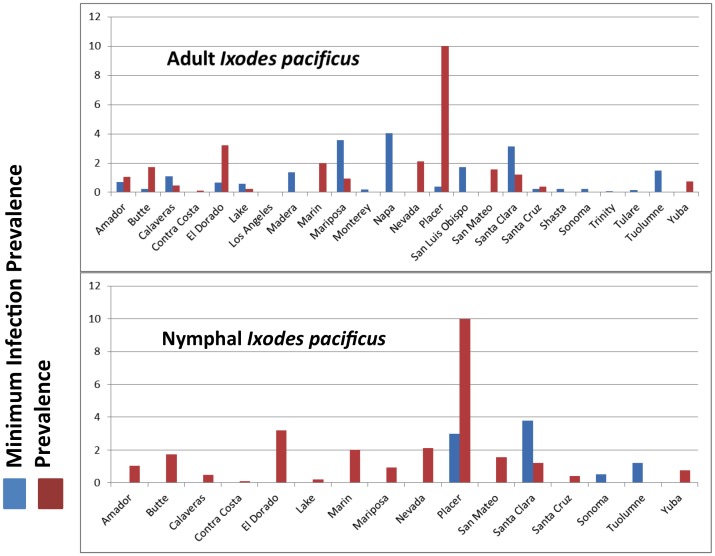
Adult and nymphal *B. burgdorferi* sl pooled results (minimum infection prevalence) and individual tick results (prevalence), 2000–2012.

**Figure 4:**
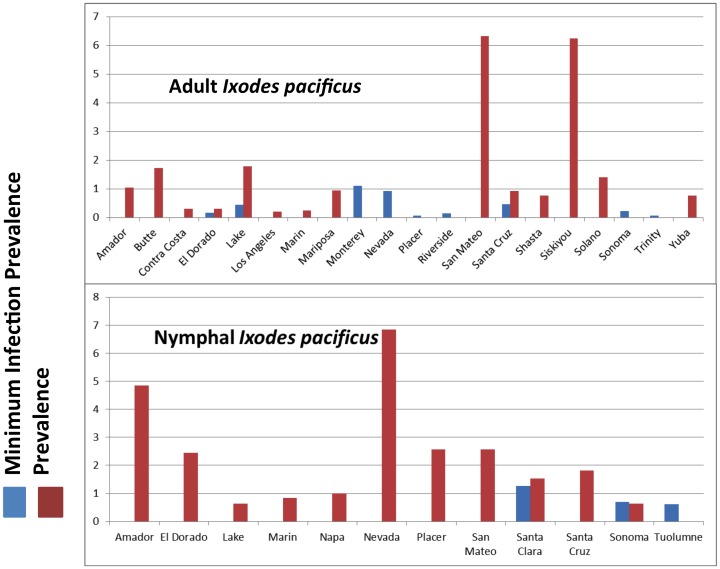
Adult and nymphal *B. miyamotoi* pooled results (minimum infection prevalence) and individual tick results (prevalence), 2000–2012.

While *B. burgdorferi* sl and *B. miyamotoi* were detected in *I. pacificus* in those regions of California where this tick species is abundant, there is variation in overall prevalence of both agents (Figure 2). *Borrelia burgdorferi* sl was more prevalent in northern and central coastal California and in the Sierra Nevada foothills: the highest MIP for *B. burgdorferi* sl in adult ticks was reported from Napa (4.04), Mariposa (3.57), Santa Clara (3.12), San Luis Obispo (1.74), and Tuolumne (1.5) counties. The highest prevalence was found in Placer (10.0), El Dorado (3.2), Nevada (2.12), San Mateo (1.58), and Santa Clara (1.2) counties (Figure 3). Similarly, *B. miyamotoi* was also highly prevalent in northern and coastal regions as well as the Sierra foothills: the highest *B. miyamotoi* MIP of adults was recorded in Monterey (1.1), Nevada (0.92), Santa Cruz (0.47), Lake (0.44) and Sonoma (0.22) counties and highest prevalence of *B. miyamotoi* in adults was recorded in San Mateo (6.33), Siskiyou (6.25), Lake (1.78), Butte (1.72), and Solano counties (1.41) (Figure 4).

Few *B. burgdorferi* sl and *B. miyamotoi* positive ticks were detected in southern California during this study. Despite testing over 5,000 adult ticks from Los Angeles County, only one *B. burgdorferi* sl positive tick was detected from Catalina Island. Similarly, Riverside County had no *B. burgdorferi* sl infected ticks detected and only two positive for *B. miyamotoi* despite over 1,600 adult ticks tested.

Similar to adult ticks, *B. burgdorferi* sl in nymphs was more commonly detected in northern California and Sierra foothill counties with the highest recorded *B. burgdorferi* sl prevalences in nymphal *I. pacificus* from Placer (10.0), Nevada (2.13), Butte (1.72), San Mateo (1.58), and Santa Clara (1.2) counties. For *B. miyamotoi*, the counties with the highest prevalence in nymphs were Nevada (6.84), Amador (4.85), Placer (2.56), San Mateo (2.56) and El Dorado (2.44) counties. All nymphal ticks were tested individually (prevalence results only). No nymphal *I. pacificus* tested positive for *B. burgdorferi* sl or *B. miyamotoi* from southern California, a region where collection of this stage is difficult.

## Discussion

This 13-year comparative study documents both *B. burgdorferi* sl and *B. miyamotoi* prevalences in *I. pacificus* ticks over a large geographic region and denotes the potential relative risk of contracting these two *Borrelia* human pathogens from nymphal and adult tick bites. These data suggest a similar risk of exposure to *B. miyamotoi* as to *B. burgdorferi* sl from adult ticks in western North America. Moreover, there is a higher risk of acquiring *B. burgdorferi* sl than *B. miyamotoi* from nymphal ticks. Both *B. burgdorferi* sl and *B. miyamotoi* are found in many regions of California but are most abundant in the north coastal and foothill regions of the state. This is the first study to provide a comprehensive estimate of entomologic risk for *B. burgdorferi* sl and *B. miyamotoi* over a large region of western North America as most studies have focused on only the region of California considered to be endemic for Lyme disease (north-western portion of state).

*Borrelia miyamotoi* is now recognized as a human pathogen in Europe and the eastern United States. One of the primary objectives for this study was to assess *B. miyamotoi* prevalence in *I. pacificus* nymphs and adults, the two stages of ticks that commonly bite people in western North America. Soon after being described in ticks in the eastern United States [8], CDPH-VBDS detected *B. miyamotoi* in California in pools of adult *I. pacificus* collected in 2000 from Sonoma, Monterey, and San Luis Obispo counties [17]. It was subsequently detected in *I. pacificus* collected in Mendocino County [18] and more recently in San Mateo and Santa Clara counties [19]. While *B. miyamotoi* has been detected in the white-footed mouse, deer, and wild turkeys in other parts of North America, this spirochete has not been detected in potential vertebrate reservoirs in California – future research is needed to understand the transmission ecology of *B. miyamotoi* in California.

In North America, seven genomospecies have been identified within the *B. burgdorferi* sl complex, including *B. burgdorferi* ss, *B. americana*, *B. andersoni*, *B. bissettii*, *B. californiensis*, *B. carolinensis, B. kurtenbachii,* and *B. garinii* [42]–[47]. The life-cycles of these *Borrelia* species are complex and involve members of the hard tick genus *Ixodes* and numerous vertebrate reservoirs [48]. To date, only *B. burgdorferi* ss, *B. americana, B. bissettii,* and *B. californiensis* have been described in *I. pacificus* from California, along with various uncharacterized *Borrelia* species [29], [42], [43].

Lyme borreliosis is the most commonly reported vector-borne disease in North America [49]. Thus far, a total of 18 *Borrelia* genomospecies have been described in Ixodid ticks worldwide [50], with the causative agent of Lyme borreliosis being designated as *B. burgdorferi* ss and other closely related species composing the *B. burgdorferi* sl complex. In the western United States, *I. pacificus* is the only tick species that transmits *B. burgdorferi* ss to humans, and the nymphal stage is the primary vector [51]. *Ixodes pacificus* is widely distributed in the far west from southern British Columbia to northwestern Baja California and as far east as Utah [52]. In California, *I. pacificus* has been recorded in 56 of 58 counties (CDPH unpublished results).

*Borrelia miyamotoi* belongs in the relapsing fever group, a separate clade from *B. burgdorferi* sl [30]. While relapsing fever group *Borrelia* are predominately associated with argasid ticks (soft ticks) such as *Ornithodoros* species, *B. miyamotoi* is the only relapsing fever group spirochete associated with ticks in the *Ixodes ricinus* species complex. In California, *B. hermsii* is the most commonly reported relapsing fever group pathogen, with an average of eight human cases reported per year [17]. This agent is transmitted to people by soft ticks, *Ornithodoros hermsi*, typically in rodent-infested mountainous cabins. Other relapsing fever group *Borrelia* species in California include: *B. parkeri*, transmitted by *O. parkeri,* primarily in California’s Central Valley, *B. turicatae*, transmitted by *O. turicatae*, and *B. coreaceae*, transmitted by *O. coriaceus*. Other than a handful of cases of *B. parkeri* in the Central Valley in the early 1900 s [53], none of the later species are typically associated with human infection in California.

The distribution of *B. miyamotoi* in California appears to mirror the range of *I. pacificus* and similar to *B. burgdorferi* sl, it is most prevalent in north-coastal and foothill regions of California where ticks are associated with hardwood conifer woodland habitats [26], [54]. In most counties in northern California, and often within the same collection sites, both *B. burgdorferi* sl and *B. miyamotoi* were detected. Results from this study suggest that although *B. miyamotoi* is detected in *I. pacificus* from diverse regions of California, both *B. miyamotoi* and *B. burgdorferi* sl have lowest prevalence in southern California. This may parallel the findings of a lower density of infected nymphs (DIN) in south-eastern North America and thus a lower risk of exposure to Lyme disease [55]. A recent study of over 2,000 adult *I. pacificus* in Los Angeles County found a similar low prevalence of *B. burgdorferi* sl, with only 0.04% *I. pacificus* positive for the human pathogen, *B. burgdorferi* ss [29]. Nymphal ticks are difficult to collect in southern California, likely due to environmental conditions, and thus few nymphs were tested from southern California in this study as well as in previous studies [29].

In this study, ticks were tested both in pools as well as individually. When infection prevalences are low, e.g., 1–5%, expected results for both approaches would be comparable. Here, the individual testing approach yielded a higher prevalence of *Borrelia* spp. in ticks than those tested in pools. This result may not necessarily be reflective of testing ticks in pools but could be because a higher proportion of ticks post-2007, when more ticks were tested individually, were from northern and foothill regions, as compared to southern California.

Unlike *B. burgdorferi* ss, which is not transmitted from infected adult female to eggs, *B. miyamotoi* is transovarially transmitted, thus larval ticks may be infected [8], [13], [25]. Furthermore, ixodid ticks can be considered a reservoir for this agent and *B. miyamotoi* does not need to rely on vertebrate reservoir hosts to maintain local infection prevalence. Previously reported *Borrelia* positive *I. scapularis, I. pacificus,* and *I. ricinus* larvae, detected by direct or indirect fluorescence antibody tests, were most likely detections of *B. miyamotoi* [25]. DFA-positive larvae from field-collected female *I. pacificus*, previously attributed to *B. burgdorferi*, were likely infected with *B. miyamotoi* [25], [56]. Furthermore, *B. miyamotoi* is difficult to culture and *Borrelia* obtained from larval ticks have been uncultivable [56]. Additional transmission studies with *B. burgdorferi*-infected laboratory *I. pacificus* colonies showed no evidence of transovarial transmission [25], [57]. While the infection of larvae with *B. miyamotoi* suggests human risk from larval tick bites, *I. pacificus* larvae are rarely reported as an ectoparasite of people (CDPH, unpublished results) [58].

With improved molecular detection methods, there is interest in cataloging the bacterial and viral communities within ticks and other biting arthropods, as some of these may be potential human pathogens [48], [59]. Out of approximately 800 adult *I. pacificus* from seven northern California counties, tested with 18 primer pairs in a combined PCR with electrospray ionization mass spectrometry (Ibis Biosciences), the prevalence of *B. burgdorferi* sl and *B. miyamotoi* was found to be similar to this study; in addition, *Rickettsia* and *Anaplasma* were detected among other bacteria and endosymbionts (Crowder, C and Eshoo, M, personal communication). Similarly, with increasing use of multiplex assays, there is interest and capabilities to detect co-infections in ticks. *Ixodes scapularis* adult ticks have been found to harbor co-infections of *B. burgdorferi* sl and *B. miyamotoi* in New York state and Canada [60], [61]. In our study, only one nymph from Marin County tested positive for both *B. burgdorferi* sl and *B. miyamotoi.*

Although *B. burgdorferi* sl group spirochetes were not further characterized to genomospecies here, it is likely that many positive ticks from California may harbor *B. bissettii, B. americana, B. andersonii,* or *B. californiensis* in addition to *B. burgdorferi* ss [45]. The diversity of *Borrelia* species in California *I. pacificus* should be taken into account when testing ticks for *B. burgdorferi* sl for surveillance purposes; if testing methods are not specific enough, the prevalence of *B. burgdorferi* ss in a tick population may be overestimated. Furthermore, additional analyses regarding the genomospecies should be taken into account to help elucidate the complex vector-reservoir-*Borrelia* strain ecology in California.

In California, 0.8% of adult and 1.4% of nymphal *I. pacificus* were infected with *B. miyamotoi*, suggesting similarly low risk of exposure to this spirochete for each stage. This is similar to surveillance results in Europe where the prevalence in adult and nymphal ticks collected in Estonia was estimated at 0.8% and 1%, respectively [15]. In comparison, the risk of acquiring *B. burgdorferi* ss is higher following a nymphal versus adult *I. pacificus* bite in California [49]. As determined by this study, statewide estimates of *B. burgdorferi* sl infection of 3.3% in nymphs and 0.7% in adults support this higher risk. Results presented here suggest comparable risk of transmission for *B. burgdorferi* sl and *B. miyamotoi* following an adult *I. pacificus* bite; however, the risk of exposure is 2.5 times higher for *B. burgdorferi* sl than for *B. miyamotoi* following a nymphal *I. pacificus* bite in California. Although this study includes a large sample size of ticks over a large geographic region, sampling was not conducted using methods such as a grid to facilitate calculation of index of risk such as density of infected nymphs (DIN) [55]. Thus, it should be noted that relative prevalences of these *Borrelia* may differ locally and temporally.

Laboratories that test ticks removed from humans or test ticks as part of a regional risk assessment program in western North America should ensure that their tests are specific for *B. burgdorferi* ss so as to not inflate risk estimates for Lyme borreliosis. Furthermore, due to the increased interest in diverse potentially pathogenic *Borrelia* species, including those within the *B. burgdorferi* sl group, more specific characterization may also prove to be informative when describing human risk for emerging tick-borne pathogens. With the knowledge that *B. miyamotoi* is present in California ticks, at a comparable prevalence to other parts of the world where human cases of *B. miyamotoi* have been reported, physicians should keep tick-borne relapsing fever as part of their differential diagnosis when evaluating patients with compatible disease following an *I. pacificus* bite and if suspected, contact their infectious disease specialist or local health officer for guidance in testing. Furthermore, these results highlight the continuing need for the general public to be familiar with tick bite prevention measures to avoid exposure to the diverse pathogens ticks carry.

## Supporting Information

Table S1 Collection Locations for *Ixodes pacificus.*
(XLSX)
